# Service user experiences of using internet-based testing for sexually transmitted infections (STIs): a qualitative study

**DOI:** 10.1136/sextrans-2024-056228

**Published:** 2024-05-31

**Authors:** Tommer Spence, Frances Griffiths, Jonathan Ross

**Affiliations:** 1 Institute of Epidemiology and Health Care, UCL, London, UK; 2 Warwick Medical School, University of Warwick, Coventry, UK; 3 Whittall Street Clinic, University Hospitals Birmingham NHS Foundation Trust, Birmingham, UK

**Keywords:** SEXUAL HEALTH, QUALITATIVE RESEARCH, HEALTH SERVICES RESEARCH

## Abstract

**Objectives:**

Internet-based testing for sexually transmitted infections allows individuals to order a self-sampling kit online, send samples to a central laboratory and receive their results electronically, reducing the need to attend a clinic unless for treatment. Its usage has grown rapidly in many high-income countries, such as England, where it now accounts for 44% of tests within the National Chlamydia Screening Programme. However, there is limited data on the experiences of service users, which may offer insights into low uptake and poor return rates among some high-incidence populations.

**Methods:**

Participants were recruited via sexual health clinics and the website of an internet-based testing service. Purposive sampling was used to ensure a diversity of genders, sexualities and ethnic backgrounds were included. Semistructured interviews were conducted by phone, email and messenger services and explored participants’ perceptions and experiences of both internet-based and clinic-based testing. Data underwent thematic analysis.

**Results:**

We interviewed 17 participants. Internet-based testing appealed to many due to the privacy and convenience it offered over clinic-based testing. Although most were positive about their experience of internet-based testing, many found the process of finger-prick blood sampling extremely challenging and this contributed to concerns from some participants that test results may be inaccurate. A minority of participants missed the opportunity that clinic-based testing offered to discuss symptoms or concerns with staff. Participants overwhelmingly found the process of receiving test results by short message service (SMS) acceptable and preferable to alternatives.

**Conclusions:**

Internet-based testing is viewed positively by most users but uptake may be improved if providers emphasise the privacy and convenience it offers, as well as the accuracy of self-sampling. Providers should also consider measures to address user concerns around blood sampling and access to specialist advice.

**WHAT IS ALREADY KNOWN ON THIS TOPIC**

- The use of internet-based testing is increasing in many countries and surveys of users indicate it is generally acceptable; however, there is a limited understanding of the service user experience and how this can be improved.

**WHAT THIS STUDY ADDS**

- This is one of the first qualitative studies exploring experiences of the entire internet-based testing pathway, using a diverse sample of service users. We found that internet-based testing appealed due to its perceived convenience and privacy, although many service users found it challenging to self-sample blood and some missed the opportunity to discuss concerns with staff.

**HOW THIS STUDY MIGHT AFFECT RESEARCH, PRACTICE OR POLICY**

- Providers can be confident that users like internet-based testing but need to consider how they can address concerns about blood sampling and ensure users are able to access specialist advice when needed. Further qualitative research should focus on populations with low uptake of internet-based testing.

## Introduction

**Figure 1:**
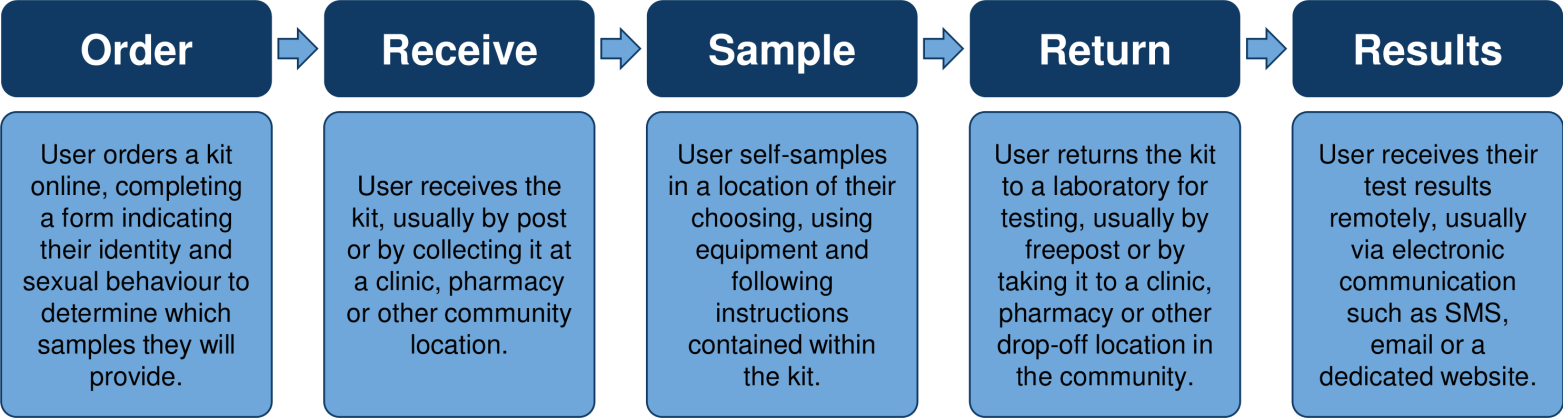
Overview of the internet-based testing process.

Internet-based testing is an alternative to testing for sexually transmitted infections (STIs) in face-to-face settings, such as sexual health clinics. Although variations exist between services, they typically involve users ordering a self-sampling kit online, which they return to a laboratory for testing before receiving their results remotely.^1 2^ Common media for results delivery include short message service (SMS), email and websites, and users who receive an STI diagnosis are typically directed to a face-to-face service for contact tracing and treatment—although entirely online follow-up services are also emerging.^1–3^ Figure 1 outlines the internet-based testing process.

Usage of internet-based testing in England has risen considerably over recent years. The number of internet-based tests conducted within the National Chlamydia Screening Programme (NCSP) was first recorded in 2015, when the figure was 79 736 (5% of total tests). This rose to 270 500 (20% of total tests) 4 years later, despite a fall in the total number of tests.^4^ Restrictions on face-to-face services during the COVID-19 pandemic further accelerated uptake of internet-based testing; although no longer a direct comparison, as the NCSP now only includes young women, 44% of tests in 2022 were accessed online.^5^

Existing research has found high levels of satisfaction with, and uptake of, internet-based testing. Surveys of users in England and the USA have repeatedly found that upwards of 80% would access it again, feel it is easy to use and consider it an acceptable service.^6–9^ Demand has been high in areas that have introduced internet-based testing, such as Birmingham, England; by the end of its first year, 33% of the service’s STI tests were accessed online, and this had risen to 50% by the beginning of its third year.^2 10^ However, research in multiple areas of England has also shown that uptake of internet-based testing varies across population groups, with users being disproportionately young, female, of white ethnicity and living in less deprived areas.^2 10–13^ One large internet-based testing service in England found that approximately half of ordered kits were not returned, with half of returned kits having insufficient samples for a full STI screen to be conducted.^2 10^ Studies in England and France have found that men who have sex with men (MSM) and people affected by deprivation are less likely to return kits.^11 14^

Understanding the discrepancy between positive survey results and the reality of service usage requires studies that explore user experience. There have been three studies undertaken in the UK which sought to do this through collecting qualitative data. Gibbs *et al* reported the perspectives of internet-based testing users on the communication of test results, finding that users perceived it as private and that they valued the autonomy it gave them in choosing when and where to access their results.^15^ Middleton *et al* explored experiences of using internet-based testing among people with mild learning disabilities, finding that kits were appealing but considerably challenging for this population to use.^16^ Ahmed-Little *et al* included a free text question in a survey of internet-based testing users, but responding to it was optional.^6^ They found that internet-based testing was acceptable to users, due to its perceived accessibility and anonymity, although some faced difficulty self-sampling blood.

This study expands existing qualitative evidence by interviewing a diverse sample of those who have used internet-based testing, about the entirety of their experience from ordering a test kit to receiving their results. It formed one part of a mixed methods PhD research project exploring service user perceptions and experiences of internet-based testing.

## Methods

### Research setting

Umbrella is a National Health Service (NHS)-delivered sexual health service in Birmingham and Solihull, one of the largest metropolitan areas in England. It offers free, unlimited access to internet-based testing for residents aged 16 or over, allowing them to test for chlamydia, gonorrhoea, syphilis, HIV and—for MSM—hepatitis B.^2 11^ Umbrella also runs face-to-face sexual health clinics throughout the area.

### Sampling and recruitment

We used quota sampling to ensure participant demographics reflected those of the local population, based on the most recent census data from Birmingham, and included populations which experience relatively high incidence of STIs. As sexual orientation was not included in the census data, the percentage of MSM was based on findings from the most recent National Survey of Sexual Attitudes and Lifestyles.^17^ Quotas were minimums of 22.5% South Asian, 9% black, 15% aged 16–24 years and 2.6% MSM. Quotas were initially set to ensure equal numbers of male and female participants, with non-binary or intersex people included irrespective of their sex. This quota was revised down during data collection to at least one-third being male or female, due to low a participation rate from male respondents and emerging data saturation. The target sample size was initially set at 20, as this was likely to be sufficient to obtain data saturation while also being large enough to enable the quota sampling. Recruitment ceased before this once saturation was apparent.

Participants were recruited via information provided on posters in two Umbrella clinics, both in Birmingham city centre, and via a web link at the end of the order form for internet-based testing kits. The recruitment information invited potential participants to take part in interviews exploring how people access testing for STIs and that participants would receive a £20 voucher. Those interested were invited to complete an online form with their name, contact details, age, sex, ethnicity and sexual orientation; all questions were optional. Respondents were emailed with the participant information sheet and asked to confirm participation within 1 week. This was done sequentially by the time of completion of the online form unless their demographic information would have prevented one of the quotas from being met, in which case they were not emailed. If no response was received, another respondent was contacted in their place.

Participants were eligible for inclusion if they were aged between 16 and 60, could speak English and had accessed STI testing via Umbrella; this was confirmed during the consent process at the start of the interview. Participants completed an online consent form before the interview, with consent being confirmed again verbally or in writing at the start.

### Data collection

Participants were given the choice of being interviewed by email, messenger, phone or Skype. The first two of these options were conducted asynchronously. Remote interview methods were used for both methodological and practical reasons. Prior research has found that participants can prefer talking about stigmatised topics in an online setting and that giving participants the agency to choose the communication medium ensures they can pick the method which makes them feel most comfortable.^18–20^ As participants could be based anywhere in Birmingham and Solihull, conducting interviews remotely also reduced travel time and costs, removed the logistical requirement of arranging a suitable location and eliminated any safety risk posed by meeting participants face-to-face.^18 21 22^

Semistructured interviews were conducted between February 2019 and February 2020 by TS, a white, male research student. Participants were asked the reasons behind their decision to access STI testing on this occasion, why they had chosen to do so via internet-based testing and their experience accessing the service, including experiences of self-sampling and receiving results. They were asked similar questions about previous experiences of testing, including their first time accessing STI testing and when they had accessed testing via a different channel (such as via a face-to-face sexual health clinic) where relevant. Participants were also asked for their overall impressions of the modes of testing they both had and had not accessed, and of potential alternatives to service provision, such as receiving test results by email.

The most popular communication medium for being interviewed chosen by participants was messenger, with six interviews taking place via WhatsApp and one via SMS. Six participants chose to conduct their interview by email and four chose to do so by phone. Phone interviews lasted between 37 and 47 min and were audio recorded and transcribed verbatim. Email and messenger exchanges took place over multiple days and the text was used directly as transcripts.

### Analysis

Transcripts were imported to NVivo V.12 software, where they underwent an adapted version of Braun and Clarke’s thematic analysis.^23^ We chose this method as our starting point due to its accessibility, proven applicability to applied health research and suitability to inform policy and practice; an anticipated outcome of this study.^24^

The transcripts were checked for accuracy and reread for familiarity before initially being coded close-to-text so that codes would retain as much of the initial meaning as possible. These codes were then amalgamated into initial themes which captured the meaning of multiple codes. This process continued to develop higher-order themes which captured the key findings across the dataset. All coding and analysis were undertaken by TS and reviewed at regular analysis meetings with FG and JR.

## Results

### Participants

Out of 834 people who expressed interest in the research, 109 were invited by email to participate. Our quota sampling resulted in interviews being arranged with 18 people. One was found to be ineligible at the start of the interview so 17 interviews were completed.

Participant ages ranged from 18 to 32, with 47% identified as white, 24% as black, 18% as Asian and 12% as mixed. Six participants were male and 11 were female. One male participant identified as bisexual, two identified as gay and the remainder—including all female participants—identified as straight.

### Themes

We identified five themes, which are set out narratively below. Illustrative quotes are reported in table 1.

**Table 1:** Summary of themes with illustrative quotes

Theme	Illustrative quote	Participant demographics
Convenience	“I noticed that [internet-based testing kits] were available from the first time I went on the website but didn’t truly consider it as an option until I realised it wouldn’t be so easy getting a walk-in appointment and that booked appointments appeared to have to be 3 or 4 weeks booked in advance.”	Black, heterosexual male, 20–24
	“The self-sampling kits are quicker than going to a clinic, as often the waiting times at the clinic are really long, even if you have pre-booked an appointment. I can do the self-sampling kits in my own time.”	White, heterosexual female, 25–29
	“I don't own a car and I work full time and often have things going on in the evenings. I also travel a lot for work so it’s not always easy for me to be in Birmingham.”	Mixed, heterosexual female, 30–34
Privacy	“I think going to the clinic is very personal and there’s always that feeling of hoping to not see somebody you know because you’re embarrassed.”	White, heterosexual female, 20–24
	“[I don’t like] having to give your full name when you book in at reception. You get a code which I presume they should use but they still ask for your name. Then the nurse will call you by your full name […] I assume everyone else is ‘Facebook stalking’ all hot guys as their names get called out.”	White, gay male, 30–34
	“It definitely feels more private to get a box in the post than have to go in and ask in a pharmacy […] just has the potential to be a bit awkward I suppose, for example, if there’s a queue of people around.”	White, heterosexual male, 30–34
Obtaining samples for testing	“I’m likely to feel like I’ve carried it out incorrectly and may have inaccurate results.”	Black, heterosexual female, 16–19
	“Upon attempting to carry out the blood sample I became very dizzy and passed out. After my experience I would never order another kit.”	Asian, heterosexual female, 25–29
	“On my most recent visit I found the nurse who took the blood tests/samples wasn’t particularly professional, she mumbled a lot and wasn’t clear about what she was doing/why, but usually this experience is fine so I feel this was just a one-off.”	White, heterosexual female, 25–29
Loss of interaction with clinic staff	“Prefer getting tested in a clinic as I trust the staff and their expertise […] there’s also a comfort element in that they can answer any worries or questions I’ve got, and if there are any worrying visual symptoms then they can look and diagnose/reassure as required.”	White, heterosexual female, 30–34
	“Last time I was at the clinic I was also diagnosed with pelvic inflammatory disease which they discovered by a quick examination of the tummy area […] I would not have known without going to the clinic.”	White, heterosexual female, 20–24
	“They were asking questions about how many units of alcohol I’m drinking per week and how many times I’m having unprotected sex if I’m under the influence of alcohol and stuff like that, so I think going to the clinic made me aware about the risks that I’m taking.”	Asian, gay male, 16–19
Communication of results	“I may not always be available for a phone call so it may require having to call back at certain times & get through to someone, therefore delaying when I get my results. A letter would have to come home and someone else may happen to open it. Logging onto a website wouldn’t be too bad but I like receiving a text and then it [being] over with.”	Black, heterosexual female, 16–19
	“[SMS is] more confidential […] you don’t want everybody to know what’s happening to you and then not everybody can access the information.”	Black, heterosexual female, 20–24
	“I’ve always kept the NHS no reply text messages on my phone in case a sexual partner wants to see proof of my testing.”	Asian, gay male, 16–19
	“[The SMS said] the rest of the results would be coming later and I feel that made me panic. I thought, ‘Why didn’t I get all of them on one? Was something wrong? Why send the rest of the results later on?’”	Black, heterosexual female, 20–24

### Convenience

Almost every participant who accessed internet-based testing stated that they did so, at least in part, because it was a more convenient option than testing in a sexual health clinic or other face-to-face setting. One reason for this was the limited number of appointments available at Umbrella sexual health clinics. Participants who wanted to attend a clinic often faced a wait of multiple weeks. Participants opted to use internet-based testing to avoid long waits in the clinic if they could get an appointment. This was a major barrier for participants who would have to take time off work to attend. Participants saved travel time by opting for internet-based testing and chose to have their kit delivered by post to avoid the need to collect it. Convenience was a particularly strong influence on access among male participants.

### Privacy

Privacy concerns led many participants to use internet-based testing. There was a widely held perception that participants’ privacy was undermined by attending a clinic, for example through the risk of seeing someone they knew in the waiting room. Even participants who claimed that they did not feel any embarrassment about attending a clinic mentioned that they were aware of others feeling this way. Concerns around privacy in clinics were particularly prominent among participants who identified as MSM. Privacy concerns also influenced how some participants accessed internet-based testing, although this manifested in different ways. For example, one participant reported ordering his kit to a pharmacy, to avoid it being found by family members in the post, while another preferred ordering it by post to avoid having to interact with other patients in a pharmacy.

### Obtaining samples for testing

Participants expressed concern about making errors when self-sampling or about their samples being affected in transit during the postal return process, thereby compromising their test results. Many participants found self-sampling blood for HIV and syphilis testing prohibitively challenging. There were few concerns expressed about obtaining urine samples or vaginal swabs. One participant reported returning his kit with only these samples, foregoing the HIV and syphilis tests, as he had found finger-prick blood sampling so difficult in the past. Although some participants had negative experiences with sampling in clinics, they viewed these as more insignificant or anomalous than their sampling difficulties with internet-based testing.

### Loss of interaction with clinical staff

Although most said it would not dissuade them from accessing internet-based testing if they saw it as the more convenient option, around a third of participants spoke positively about interacting with clinical staff while testing in face-to-face settings and some missed this when using internet-based testing. Most participants who mentioned this said that it would only apply if they had concerning symptoms. This view was more common among female participants and for several participants was based on positive experiences of testing in face-to-face settings. One participant who had felt judged by a clinician on their previous, and first, visit to a clinic still said they felt there was value in interacting with clinic staff who may identify risks in your sexual behaviour which you are oblivious to.

### Communication of results

Participants were almost unanimous in their satisfaction with having test results communicated via SMS. As with accessing internet-based testing, this was largely driven by the perception it was a more convenient or private option. Many participants expressed specific dissatisfaction with alternative methods of results communication, such as a phone call or website, again because of lack of privacy or inconvenience. Even a participant who had received a positive test result via SMS and found the experience *“*mortifying” and “a bit of a shock”, still felt that it was the best medium for results to be delivered due to its convenience; he was concerned a more complex process of accessing results—such as logging into a website—may have put him off accessing them. The same participant stated that they felt SMS results offered a unique benefit, as his STI status was always readily available on his phone to show a prospective sexual partner. There were some criticisms of the way results were delivered, with some participants highlighting that the delivery of individual STI test results separately could cause confusion or anxiety. No participant stated, however, that their criticisms would wholly deter them from receiving their results via SMS.

## Discussion

We found that internet-based testing appeals to many participants due to the perceived privacy and convenience it offers over clinic-based testing. Although most participants were positive about their experience, many found the process of finger-prick blood sampling extremely challenging. This contributed to concerns that test results may be less accurate than samples obtained by a clinician. Some participants also appreciated the opportunity for face-to-face, clinic-based testing offered to discuss symptoms or concerns with staff and receive advice. Participants overwhelmingly found the process of receiving test results by SMS acceptable and preferable to alternatives.

Our study is one of the first to explore the entire experience of using internet-based testing via in-depth, qualitative methods. Its strengths include the highly diverse sample, which reflected both the local community and populations with the highest incidence of STIs. The study was also strengthened by most participants having experience with both internet-based testing and testing in face-to-face settings, so they were able to compare these experiences in their interviews. The study was limited by the small sample, which meant that the size of demographic subgroups was extremely small. Although data saturation was reached across our sample, a larger study would be needed to recruit sufficient participants from subgroups to ensure data saturation for these populations of interest. Participants were also only recruited from one site, limiting the applicability of the findings to internet-based testing services which operate in different ways. We were also unable to draw conclusions on which barriers are prohibitive to some people using internet-based testing, as we only recruited participants who had accessed it.

Almost every participant expressed a willingness to use internet-based testing, which is consistent with previous survey findings.^6–9^ This finding on acceptability is more emphatic than many existing qualitative studies, however, which did not include participants who had accessed real internet-based testing services, demonstrating the value of conducting qualitative research focusing on experiences of services. There is considerable overlap with previous qualitative studies on STI testing with respect to the themes which influence access, including privacy, convenience, sample accuracy and interaction with clinical staff.^25–28^

Our finding that almost every participant struggled with self-sampling blood is valuable context for existing quantitative data. Banerjee *et al*,^10^ for example, identified that only half of returned internet-based testing kits had sufficient quantities of blood for HIV testing. Studies on HIV self-testing have also found that users have lower confidence in blood-based results, compared with saliva-based HIV tests, and sometimes resort to dangerous practices in order to obtain enough blood, such as using non-medical blades in their home, in place of the provided lancets.^29^

The high acceptability of SMS for results delivery contrasts with some existing studies, such as Gibbs *et al*,^15^ which found that participants were satisfied receiving results via a website but not by SMS. This suggests that internet-based testing users are generally satisfied with whatever results delivery platform they end up using—a finding consistent with the authors’ published evidence review on the topic.^25^

There are a number of recommendations for practice which can be drawn from our findings. Providers should ensure that every aspect of internet-based testing offers convenience and privacy, as far as possible, in order to facilitate access, and that the confidentiality of the service is advertised to potential users. This could include easy-to-use websites, prompt delivery of kits and discreet packaging. The challenges reported in taking blood suggest further development of the testing kit is needed, to reduce the volume of blood required, but, in the meantime, providers should explore offering instructions in a variety of formats—including videos and vignettes—to assess whether these improve accessibility for service users. They may want to consider allowing users to opt out of blood-based STI tests, or offering alternative methods for these, such as saliva-based HIV self-testing. Our findings suggest that providers can be confident using SMS as their sole method of results delivery but may want to offer a choice for service users who would prefer an alternative method, such as email or an online portal. Providers should also try to deliver all STI test results simultaneously, to reduce anxiety or confusion among service users.

## Data Availability

No data are available.
